# Human β-Defensin 23 as a Carrier for In Vitro and In Vivo Delivery of mRNA

**DOI:** 10.3390/pharmaceutics15102477

**Published:** 2023-10-17

**Authors:** Kyoung-Ran Kim, Junghyun Kim, Seunghye Cho, Dae-Ro Ahn

**Affiliations:** 1 Chemical and Biological Integrative Research Center, Institute of Science and Technology (KIST), Hwarangno 14-gil 5, Seongbuk-gu, Seoul 02792, Republic of Korea; krkim830@kist.re.kr (K.-R.K.); junghyun8808@gmail.com (J.K.); 2 Division of Biomedical Science and Technology, KIST School, University of Science and Technology (UST), Hwarangno 14-gil 5, Seongbuk-gu, Seoul 02792, Republic of Korea; seunghye@kist.re.kr

**Keywords:** β-Defensin, mRNA delivery, peptide carrier, drug delivery

## Abstract

The successful application of mRNA therapeutics hinges on the effective intracellular delivery of mRNA both in vitro and in vivo. However, this remains a formidable challenge due to the polyanionic nature, longitudinal shape, and low nuclease resistance of mRNA. In this study, we introduce a novel mRNA delivery platform utilizing a human β-defensin peptide, hBD23. The positive charge of hBD23 allows it to form nanocomplexes with mRNA, facilitating cellular uptake and providing protection against serum nucleases. When optimized for peptide-to-mRNA (N/P) ratios, these hBD23/mRNA complexes demonstrated efficient cellular delivery and subsequent protein expression both in vitro and in vivo. Importantly, as hBD23 is human derived, the complexes exhibited minimal cytotoxicity and immunogenicity. Given its high biocompatibility and delivery efficiency, hBD23 represents a promising platform for the in vitro and in vivo delivery of mRNA.

## 1. Introduction

The introduction of messenger RNAs (mRNAs) into cells to express relevant proteins is a promising therapeutic strategy for various diseases caused by protein defects [1]. However, the susceptibility of mRNA to nuclease degradation in body fluids and its difficulty penetrating the negatively charged cell membrane due to its polyanionic nature pose significant challenges to its in vivo delivery. Therefore, reliable intracellular and in vivo mRNA delivery methods are crucial for harnessing the therapeutic potential of mRNA.

Lipid nanoparticles (LNPs) have emerged as the most successful platform for delivering mRNA therapeutics, owing to their ability to protect mRNA from degradation and facilitate its delivery to target cells in vivo [2,3,4,5,6]. This was recently demonstrated in the development of mRNA vaccines during the COVID-19 pandemic [7]. Indeed, all FDA-approved mRNA vaccines are formulated with LNPs [8]. However, LNPs have several drawbacks. The success of an LNP product largely depends on the systematic optimization of its lipidic components, which can be a complex and time-consuming process. LNPs have low mRNA payloads (approximately 5%) [9], contain a considerable amount of polyethylene glycol (PEG) that may cause adverse effects such as anaphylaxis [10], and require storage at low temperatures (approximately −20 °C with 10% *w*/*v* sucrose) [2]. Furthermore, LNP technologies are heavily protected by precedented patents, which restricts the intellectual property of other potential approaches using LNPs. Therefore, there is a high demand for alternative mRNA delivery platforms.

Cell-penetrating peptides (CPPs) are short peptides capable of crossing cell membranes and delivering cargo molecules into cells [11]. They have been used for the delivery of various therapeutic agents, including nucleic acids, proteins, and small molecules [12]. The unique properties of CPPs, such as their ability to penetrate cell membranes without causing significant damage, make them an attractive delivery method. For mRNA delivery, CPPs enriched with cationic and/or hydrophobic residues form nanoparticles with mRNA for efficient cellular uptake [13,14]. Covalently conjugated CPP on mRNA could also show a certain level of transfection efficiency [15]. The CPP/mRNA complex is a two-component formulation that can be prepared with relatively high mRNA payload and simple preparation process compared with LNPs.

However, previous research efforts on mRNA delivery using CPP have been largely focused on in vitro cellular delivery. Few studies reported CPP-mediated in vivo mRNA delivery [16]. For in vivo mRNA delivery studies, CPPs have been chemically modified or mainly utilized as a component of hybrid materials for carriers [17,18,19]. In addition, most of the previously employed CPPs for mRNA delivery have sequences derived from non-human organisms or modified from human protein sequences [13]. Human-derived CPPs with unmodified natural sequences are more desirable for biomedical applications as they are expected to have low cytotoxicity and immunogenicity. In this context, we have found that peptides derived from annexin and human β-defensins (hBDs) are CPPs that are able to intracellularly deliver various nucleic acid molecules [20,21,22]. In particular, hBD23 was employed for the lung-targeted in vivo delivery of polymeric antisense oligonucleotide (pASO) [22], illustrating that hBD23 is suitable for in vivo delivery as well as the cellular delivery of bioactive nucleic acids.

In this study, we initially investigate the potential of hBD23 as a platform for the delivery of mRNA into cells. After preparing hBD23/mRNA complexes, we examine the mRNA delivery efficiency of hBD23 and the corresponding protein expression level in cellular transfection.

## 2. Materials and Methods

### 2.1. Materials

hBD23 (GTQRCWNLYGKCRYRCSKKERVYVYCINNKMCCVKPKYQPKERWWPF) was gained by Peptron (Daejeon, Republic of Korea, purity of peptide was >95%), while 5-methoxyuridine-modified mCherry, enhanced green fluorescence protein (EGFP), ovalbumin and firefly luciferase (fLuc) mRNA were purchased from Trilink Biotechnologies (San Diego, CA, USA). Anti-ovalbumin and anti-β-actin antibody were obtained from Santa Cruz Biotechnology (Dallas, TX, USA) and Cell Signaling Technology (Danvers, MA, USA), respectively. Lipofectamine RNAiMax and mouse IL-6 ELISA kit were acquired from Thermo Fisher Scientific (Waltham, MA, USA). The endocytosis inhibitors chlorpromazine hydrochloride (CPZ), 5-(N-ethyl-N-isopropyl)-amiloride (EIPA), methyl-β-cyclodextrin (MβCD) and chloroquine (CQ) were from Sigma Aldrich (St, Louis, MO, USA).

### 2.2. Preparation of hBD23/mRNA Complex

The hBD23 and mRNA were mixed at various N/P ratios (N/P = the number of the ammonium group in hBD23/the number of the phosphate group in mRNA, 0.1, 0.2, 0.33, 1, 3, 5, 10, and 20) in 1X PBS and incubated at room temperature for 1 h under shaking. Complexation between hBD23 and mRNA (0.4 μg) was confirmed by 1% agarose gel, stained with SYBR Gold, and visualized with a gel imaging instrument (iBright™ FL1000, Thermo Fisher Scientific, Waltham, MA, USA). The complexes at N/P = 1 contain approximately 40% mRNA payload. Accordingly, the complexes at N/P ratios ranging from 0.1 to 20 contain an mRNA payload of approximately 87.1% to 3.5%, respectively.

### 2.3. Characterization of hBD23/mRNA Complex

The hydrodynamic size and surface charge of the hBD23/mRNA complex (N/P = 1:10, 1:5, 1:3, 1:1, 3:1, 5:1, 10:1 and 20:1 in 1X PBS) were measured using a Zetasizer (Malvern Instruments, Worcestershire, UK). For observing the N/P ratio-dependent size change in the dried state of hBD23/mRNA complexes (N/P = 0.1, 1, 10), atomic force microscopy (AFM, Park systems, XE-100, Suwon, Republic of Korea) was used. The samples were dropped and dried onto a mica coated with NiCl2 and dried. Images were obtained using a non-contact cantilever (PPP-NCHR, Park systems, Suwon, Republic of Korea) and analyzed using XEI software 4.3.0 from Park systems.

### 2.4. Serum Nuclease Resistance

To test serum stability, 10% fetal bovine serum (FBS) was added to naked mRNA and hBD23/mRNA (0.4 μg of mRNA, N/P ratio = 1:10, 1:1, 10:1), and the mixtures were incubated at 37 °C for pre-determined time points (0, 0.5, 1, 2, 4, 7, and 24 h). The samples were analyzed on 1% agarose gel electrophoresis at 120 V for 40 min and stained with SYBR Gold. The results were visualized using an iBright™ FL1000.

### 2.5. Cellular Uptake Study

The mCherry mRNA was fluorescently labeled using the NTP mixture containing FAM-UTP (5% of total UTP) when in vitro transcription (IVT) reaction was performed. Human embryonic kidney (HEK293) cells (5 × 10^4^) were treated with FAM-labeled mRNA (1 µg) and hBD23/FAM–mRNA complexes in serum-containing media and incubated at 37 °C for 4 h. After washing two times with ice-cold DPBS, FAM-positive cells were measured using a flow cytometer (CytoFLEX, Beckman Coulter, Palo Alto, CA, USA). A total of 5000 cells were recorded for each sample, and experiments were triplicated. To investigate the endocytosis mechanism in HEK293 cells, endocytosis inhibitors (10 μM chloropromazine (CPZ), 1 mM methyl-β-cyclodextrin (MβCD), 50 μM ethylisopropyl amiloride (EIPA), and 50 μM chloroquine (CQ)) in serum-containing media were pre-incubated with cells prior to treatment with the hBD23/mRNA complexes. Flow cytometry analysis of the samples was performed as described above.

For the visualization of intracellular mRNA delivered by hBD23, HEK293 cells (2.5 × 10^4^) seeded on a confocal dish, treated with FAM-labeled mRNA or hBD23/mRNA complexes (N/P = 0.1, 0.2, 0.33, 1, 3, 5, 10, and 20) were incubated at 37 °C for 4 h. After washing two times with PBS, cells were fixed with 4% formaldehyde solution for 10 min, and the nucleus was stained with Hoest34580. The fluorescence of HEK293 cells was visualized using a confocal laser scanning microscopy (LSM800, Cal Zeiss, Oberkochen, Germany) at the magnification of ×400.

### 2.6. Intracellular Toxicity and Immunogenicity Assay

Cell viability was assessed using a Cell Counting Kit-8 (Dojindo, Kumamoto, Japan). HEK293 cells were seeded onto a 96-well plate, cultured until 80% confluency, and treated with hBD23 (0–150 μM) and hBD23/mRNA for 24 h in DMEM containing 10% FBS. Then, WST-1 reagent (10 μL) was added to each well and then incubated for 1 h at 37 °C. The absorbance of each well was determined using a microplate reader system (VICTOR Nivo™ Multimode Microplate Reader, Perkin Elmer, Waltham, MA, USA) at 450 nm. Cell viability was normalized by the viability of PBS-treated cells. The results from triplicate experiments were analyzed by Prism software 10.0.3 (GraphPad, Boston, MA, USA). Data are presented as the mean ± standard deviation (SD).

To test the immunogenicity of hBD23 and hBD23/mRNA complexes, RAW264.7 cells were seeded on 24-well plates and cultured for 24 h. The cells were grown to 80% confluency and treated with lipopolysaccharides (LPS, 10 μg/mL), hBD23 (0–150 μM), hBD23/mRNA, or mRNA/LF RNAiMax (1.5 or 3 μL) in serum-containing DMEM (300 μL) for 24 h. The supernatants were transferred to 1.5 mL tubes and centrifuged at 10,000× *g* for 10 min at 4 °C. For the detection of interleukin-6 (IL-6), a mouse IL-6 ELISA kit (Thermo Scientific, Waltham, MA, USA) was used. The results from triplicate experiments were analyzed by Prism software (GraphPad, Boston, MA, USA). Data are presented with the mean ± standard deviation (SD).

### 2.7. Evaluation of hBD23/mRNA-Mediated Protein Expression

To evaluate protein expression efficiency, 1 µg of mCherry mRNA (mC-mRNA) or EGFP mRNA (eG-mRNA) complexed with Lipofectamine RNAiMax or hBD23 peptides (N/P = 0.1, 0.2, 0.33, 1, 3, 5, 10, and 20) were treated to HEK 293 cells seeded on a glass-bottomed 35 mm dish. After 24 h, the expression of mCherry or EGFP proteins was visualized with confocal fluorescence microscopy (LSM800, Carl Zeiss).

For quantitative analysis of protein expression, cells were analyzed by flow cytometry at 24 h after treatment with hBDF23/mRNA complexes at various N/P ratios (0.1, 0.2, 0.33, 1, 3, 5, 10, and 20). The expression efficiency was calculated by counting the number of cells expressing mCherry or EGFP.

For Western blot analysis, HEK293 cells treated with LF RNAiMax or hBD23/mRNA complexes (3 µg of ovalbumin mRNA) for 24 h were lysed with 1X RIPA buffer. Subsequently, cell lysates (20 µg) were separated on 5–12% SDS-polyacrylamide gel and then transferred to a PVDF membrane. The membranes were blocked with 5% BSA in TBS-T for 1 h at RT and incubated with primary antibodies (OVA and β-actin, 1:1000, 5%BSA in TBST) overnight at 4 °C. The membranes were then washed with TBS-T three times and exposed to secondary antibodies (anti-mouse IgG-HRP and anti-rabbit IgG-HRP, respectively. 1:10,000, 5% skim milk in TBST) for 1 h at RT. After washing with TBS-T, the membranes were developed using an ECL substrate (Super Signal™ West Pico Chemiluminescent, Thermo Fisher Scientific, Waltham, MA, USA) and visualized by iBright™ FL1000.

### 2.8. In Vivo Bioluminescence Imaging

All experimental procedures involving live animals were conducted in strict accordance with pertinent regulations and institutional protocols (Approval Number: KIST-2022-047-3). Male BALB/c mice aged 6 weeks were purchased from Orient Bio (Seongnam, Republic of Korea). The firefly luciferase (fLuc) mRNA and hBD23/mRNA complexes (m RNA: 2 μg) in PBS (50 μL) were injected into animals subcutaneously. To check the bioluminescence intensity (BLI) of mice at 3 h and 24 h post injection, mice were intraperitoneally injected with D-luciferin (Promega Corporation, Madison, WI, USA) at a dose of 150 mg/kg. Mice were anesthetized after receiving D-luciferin in a chamber with 3% isoflurane and placed on the imaging platform while being maintained on 2% isoflurane via a nose cone. Mice were imaged at 10 min post-administration of D-luciferin by an IVIS Imaging Spectrum System. Bioluminescence values were quantified by measuring photon flux (photons/second) in the region of interest (ROI) where the bioluminescence signal was emanated using the IVIS Living Imaging 3.0 software.

### 2.9. Statistical Analysis

The statistical analysis was performed using Prism 10.0.2 (Graphpad, Boston, MA, USA). Statistical significance was determined by two-way ANOVA. Statistical significance was considered at a *p*-value less than 0.05.

## 3. Results and Discussion

### 3.1. Preparation and Characterization of hBD23/mRNA Complexes

**Figure 1:**
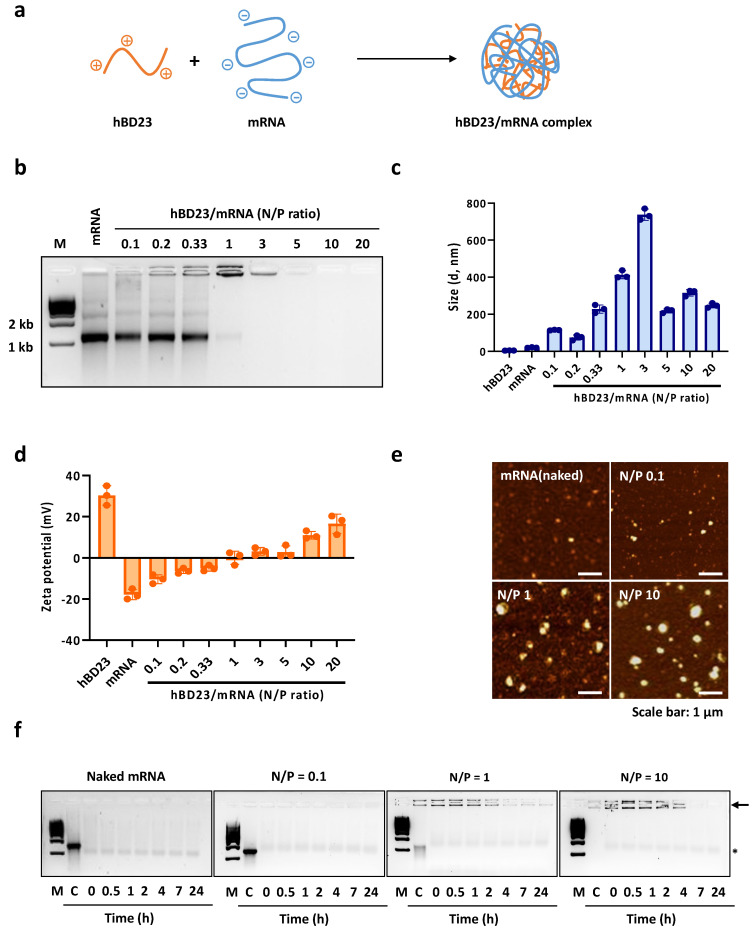
(**a**) Schematic presentation of negatively charged mRNA (mC-mRNA) complexed with positively charged hBD23. (**b**) Agarose gel electrophoresis of hBD23/mRNA complexes at various N/P ratios. M denotes the size marker. (**c**) Hydrodynamic sizes and (**d**) zeta potentials of hBD/mRNA complexes estimated by DLS. (**e**) AFM images of hBD23/mRNA complexes. (**f**) Serum stability of mRNA after complexation with hBD23 analyzed by gel electrophoresis. C denotes the control (complexes in the absence of serum). Free and complexed mRNA are indicated by an asterisk and an arrow, respectively. M denotes 1 kb size marker.

The hBD23 peptide was combined with mCherry mRNA (mC-mRNA) at varying N/P (positive charge of peptide/negative charge of mRNA) ratios (Figure 1a). The formation of hBD23/mRNA complexes was confirmed by the retarded mobility of mRNA and the disappearance of the mRNA band, which resulted from the replacement of the staining dye by the peptide in mRNA, as observed in the gel electrophoresis image (Figure 1b). The band for complexes began to appear at N/P = 0.2. The mRNA band completely vanished at N/P = 3, indicating that all mRNA was complexed with hBD23. At N/P = 10, the band for complexes also disappeared, suggesting that all staining dyes in mRNA were replaced by hBD23 within the complexes. The size of the complexes at various N/P ratios, measured using dynamic light scattering (DLS), initially increased with the addition of hBD23 and then stabilized to approximately 300 nm at high N/P (N/P > 5) ratios (Figure 1c). Due to the multiple positive charges of hBD23, the zeta potential values of the complexes became increasingly positive with higher N/P ratios (Figure 1d). The nanoscale size of complexes was also confirmed by atomic force microscopy (AFM) (Figure 1e). Consistent with DLS data, AFM images also showed the N/P ratio-dependent size variation of complexes. The observed size discrepancy between AFM and DLS may stem from the differing conditions under which measurements were taken. DLS profiles were obtained while the complexes were in solution, whereas AFM images were captured when the complexes were in a dried state. Consequently, the size pattern observed in AFM may vary from that seen in DLS, depending on the degree of dehydration. The serum stability of mRNA was examined at N/P = 0.1, 1, and 10 (Figure 1f). Gel electrophoresis images of complexes treated in the presence of 10% fetal bovine serum (FBS) demonstrated that mRNA was better protected in complexes with higher N/P ratios.

### 3.2. Cellular Uptake of hBD23/mRNA Complexes

**Figure 2:**
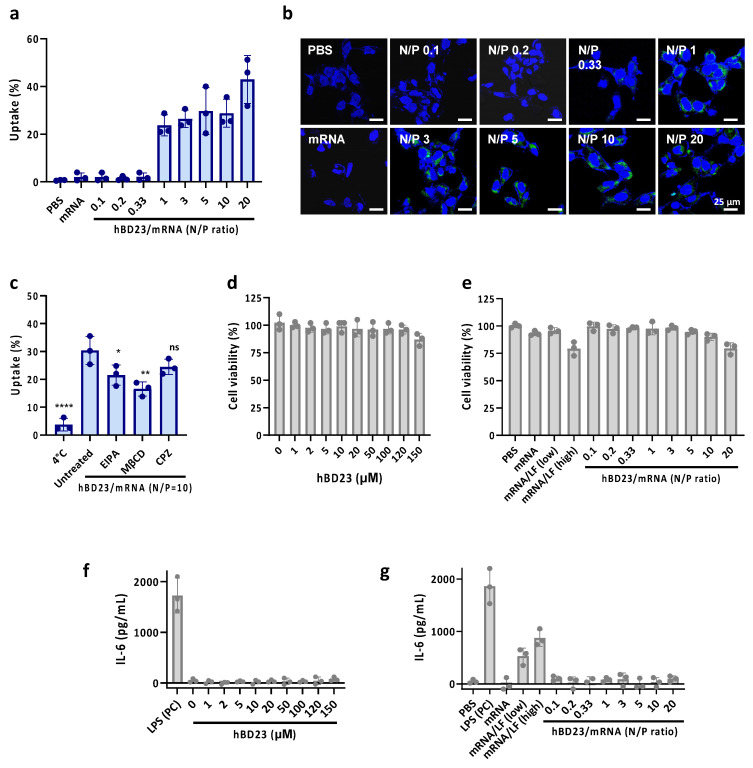
The intracellular delivery of mRNA (mC-mRNA) using hBD23 as a carrier. (**a**) Cellular uptake levels of hBD23/mRNA complexes at various N/P ratios. (**b**) Microscopic images of HEK293 cells after treatment of FAM-labeled mRNA complexed with hBD23. (**c**) Cellular uptake levels of hBD23/mRNA complexes (N/P = 10) in the presence or absence of endocytosis inhibitors. **** *p* < 0.0001, ** *p* < 0.01, * *p* < 0.05, ns (no significance) vs. untreated. Viability of HEK293 cells after treatment with (**d**) hBD23 and (**e**) hBD23/mRNA complexes. IL-6 levels released from cells after treatment with (**f**) hBD23 and (**g**) hBD23/mRNA complexes.

Following the characterization of hBD23/mRNA complexes, we estimated the cellular uptake efficiency of mRNA in the complexes. For mRNA tracking, we labeled mRNA with fluorescein (FAM) by incorporating FAM-labeled UTP during the in vitro transcription (IVT) of mRNA. Flow cytometric analysis of HEK293 cells treated with hBD23/FAM-mRNA complexes revealed significant cellular uptake levels of mRNA in the complex formed with N/P = 1 – 20 (N/P1, N/P3, N/P5, N/P10, and N/P20) (Figure 2a). Consistent with the flow cytometric data, confocal microscopic images also showed cellular uptake of the complexes (Figure 2b). The complexes were primarily localized in the cytoplasm, where protein expression occurs.

To investigate the cellular uptake mechanisms of the complexes, cells were treated with N/P1 and N/P10 in the presence of inhibitors of endocytosis pathways. The uptake efficiency significantly decreased at low temperature, indicating that N/P20 was internalized via endocytosis. The uptake level also significantly decreased in the presence of 5-(*N*-ethyl-*N*-isopropyl) amiloride (EIPA), methyl-β-cyclodextrin (MβCD), but it did not change significantly with chlorpromazine (CPZ) treatment (Figure 2c). These results suggest that macropinocytosis and caveolae-mediated endocytosis are involved in the intracellular delivery of the complexes.

### 3.3. Cytotoxicity of hBD23/mRNA Complexes

We subsequently evaluated the in vitro cytotoxicity and immunogenicity by assessing cell viability and changes in cytokine levels following treatment with the hBD23 peptide and hBD23/mRNA complexes. The viability of HEK293 cells showed negligible changes upon treatment with the peptide at concentrations ranging from 0 to 150 µM (0–15 nmol) (Figure 2d) and the complexes at N/P ratios from 0.1 to 10 (Figure 2e). Cell viability was slightly reduced to approximately 75% following treatment with mRNA complexed with lipofectamine (LF, RNAiMax) and the hBD23/mRNA complexes at N/P = 20. The level of IL-6 released from RAW264.7 cells did not significantly increase after treatment with the peptide (Figure 2f) and the complexes (Figure 2g), whereas mRNA/LF exhibited moderate immunogenicity compared with the positive control, lipopolysaccharide (LPS). These results demonstrate that both the hBD23 peptide and hBD23/mRNA complexes are biocompatible materials with low cytotoxicity and immunogenicity.

### 3.4. In Vitro Delivery of hBD23/mRNA Complexes for Protein Expression

**Figure 3:**
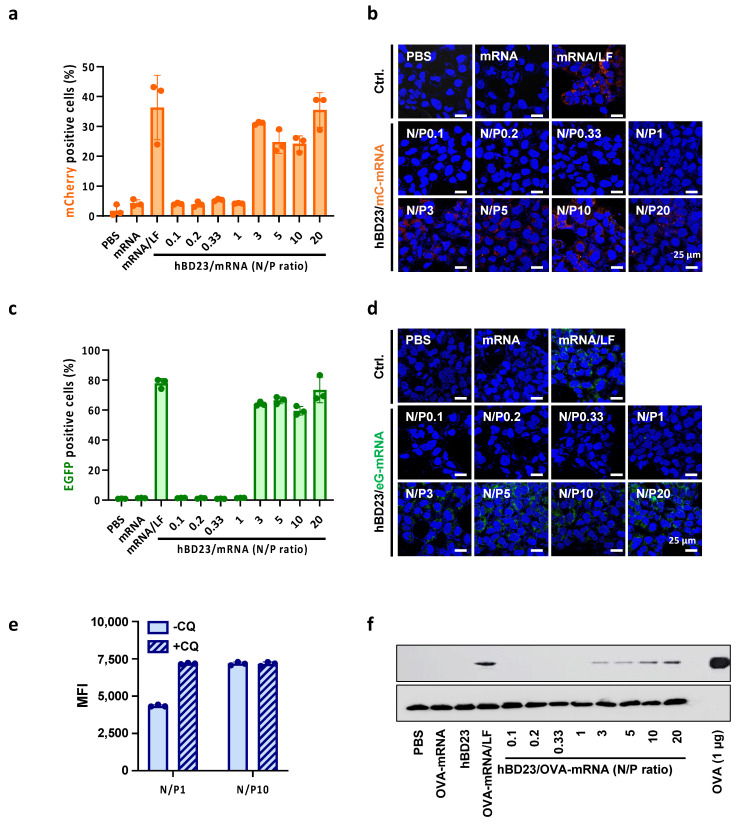
In vitro expression of protein expression by cellular delivery of mRNA using hBD23. Flow cytometric quantification of (**a**) mCherry- or (**c**) EGFP-positive cells after treatment of HEK293 cells with hBD23/mC–mRNA or hBD23/eG–mRNA complexes at various N/P ratios, respectively. Fluorescence microscopic images of HEK cells after treatment with (**b**) hBD23/mC-mRNA or (**d**) hBD23/eG-mRNA complexes at various N/P ratios, respectively. (**e**) Fluorescence intensity-based cellular uptake levels of the hBD23/mRNA complexes at N/P = 1 and 10 in the presence or absence of CQ. (**f**) Ovalbumin protein expression in HEK293 cells after treatment of the cells with hBD23/OVA–mRNA complexes at various N/P ratios.

After observing that hBD23/mRNA can be delivered into cells without significant cytotoxicity and immunogenicity, we next investigated whether the intracellularly delivered mC-mRNA could be translated to express the mCherry protein. The transfection efficiency, related to intracellular mCherry protein expression, was estimated by measuring the percentage of mCherry fluorescence-positive cells via flow cytometry. Treatment of cells with the complexes at N/P = 3–20 (N/P3, N/P5, N/P10, and N/P20) resulted in a considerable proportion of mCherry-positive cells, while the transfection efficiency by the complexes at N/P = 0.1–1 (N/P0.1, N/P0.2, N/P0.33, and N/P1) was negligible (Figure 3a). These results indicate that the N/P ratios effective for cellular uptake resulted in subsequent protein expression. The highest transfection efficiency was achieved with N/P10, which showed similar protein expression efficiency to that of mRNA delivered with lipofectamine used as the positive control. Confocal microscopic images showed cellular uptake of the complexes consistent with the flow cytometric data (Figure 3b). When cells were treated with hBD23/mRNA complexes in which mC-mRNA was replaced with EGFP mRNA (eG-mRNA), EGFP expression was observed at similar N/P ratios in both microscopic and flow cytometric analysis (Figure 3c,d). Microscopic analysis, being a qualitative method, may result in the fluorescence intensity in the imaged region being partially higher than the average value across numerous cells. However, flow cytometry, which screens several thousand cells per analysis, is more apt for the quantitative analysis of cellular uptake efficiency due to its broader sampling. Taken together, these results confirm that the intracellular expression of protein indeed results from the mRNA delivered with hBD23.

The cellular uptake level of mRNA by N/P1 did not lead to relevant translation efficiency (Figure 2a and Figure 3a). We hypothesized that an inefficient translation of mRNA of N/P1 may be due to partial endosomal entrapment. To examine this hypothesis, we measured the mean fluorescence intensity (MFI) of cells after treatment with hBD23/mRNA labeled with a pH-sensitive dye (fluorescein, FAM) via the incorporation of FAM-UTP into mRNA in the presence or absence of chloroquine (CQ), which enhances endosomal escape. As the fluorescence intensity of FAM at endosomal pH (pH = 5–6) is far lower than that at cytoplasmic pH (pH = 7.2–7.8), endosomal entrapment can be indicated by the CQ-induced increase in MFI of cells. The MFI of cells treated with the complexes at N/P = 1 was significantly increased by CQ, whereas that of the complexes at N/P = 10 was not significantly changed by CQ treatment (Figure 3e). This shows that a considerable amount of the internalized complexes at N/P = 1 failed to escape endosomes and thereby were not translated to protein.

To investigate whether the hBD23-mediated intracellular delivery of mRNA for subsequent protein expression could be replicated with mRNA for the expression of ovalbumin (OVA), a conventional model antigen for vaccine studies, we prepared hBD23/mRNA complexes with ovalbumin mRNA (OVA-mRNA). Following the treatment of HEK293 cells with hBD23/OVA-mRNA complexes at various N/P ratios, the expression of OVA protein was confirmed using Western blotting. As depicted in Figure 3f, OVA was expressed at N/P = 3–20. The highest expression level, slightly lower than that of the positive control (OVA-mRNA/LF), was observed at N/P = 20. These results suggest that hBD23 can serve as an efficient mRNA delivery platform for the expression of various proteins in cells when complexed with the cargo mRNA at an optimized N/P ratio.

### 3.5. In Vivo Delivery of hBD23/mRNA Complexes for Protein Expression

**Figure 4:**
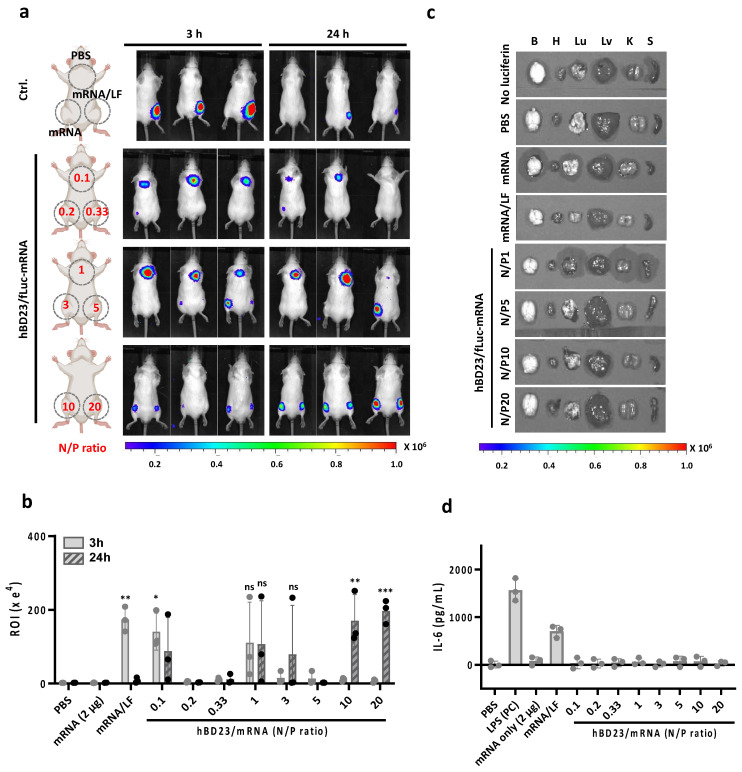
In vivo protein expression by mRNA delivery using hBD23. (**a**) In vivo imaging of fLuc protein after subcutaneous injection of hBD23/fLuc–mRNA complexes at various N/P ratios indicated with red colored text. The injection sites were indicated with dotted circles on the schematic illustration of mice. (**b**) Quantification of the in vivo bioluminescence of fLuc at the injection sites. *** *p* < 0.001, ** *p* < 0.01, * *p* < 0.05, ns (no significance) vs. PBS. (**c**) Ex vivo imaging of fLuc protein in major organs such as brain (B), heart (H), lung (Lu), liver (Lv), kidney (K), and spleen (S) after subcutaneous injection of hBD23/fLuc–mRNA complexes at various N/P ratios. (**d**) IL-6 levels in blood collected from mice injected with hBD/fLuc–mRNA complexes. LPS was used as a positive control (PC).

After establishing that hBD23 can facilitate the intracellular delivery of mRNA for subsequent protein expression at the in vitro cellular level, we explored its potential as a carrier for the in vivo delivery of mRNA. For in vivo protein expression imaging, we used firefly luciferase mRNA (fLuc-mRNA) as a cargo of the peptide carrier. We subcutaneously injected hBD23/fLuc-mRNA complexes, prepared at various N/P ratios, into the dorsal area of BALB/c mice. The fLuc protein expression was analyzed by IVIS, which is an in vivo imaging instrument. Prior to imaging, we intraperitoneally injected D-luciferin into the mice. The bioluminescence images of mice obtained at 3 h and 24 h post-injection revealed significant expression levels of fLuc following the injection of complexes formed with N/P = 0.1, 1, 10, and 20 (Figure 4a). At 3 h, the highest expression level was observed in the area treated with complexes at N/P = 0.1 (Figure 4b). The fLuc expression level of N/P0.1 was similar to that of the positive control, fLuc-mRNA/LF. At 24 h, the highest expression level was observed in the area treated with complexes at N/P = 10 and 20. This relatively late protein expression could be due to the slow release of mRNA from the complexes at such high N/P ratios. Although the average protein expression level seemed considerably high at N/P = 1, it was not statistically significant. However, the luminescence intensity of fLuc at some of the N/P ratios suitable for in vitro protein expression (N/P = 3, 5) was lower than those at the optimal N/P ratios for in vivo imaging at both 3 and 24 h. These results suggest that the in vivo evaluation of mRNA delivery platforms is critical to potential viability, as in vivo mRNA delivery efficiency for protein expression is unpredictable with in vitro mRNA delivery efficiency. It is also worth noting that the in vivo transfection efficiency of LF is generally lower than that of LNP. Consequently, the in vivo transfection efficiency of hBD23 is anticipated to be less than that of LNP.

No fLuc expression in all major organs after the injection of hBD/mRNA complexes supports that systemic toxicity may not be induced by the subcutaneous injection of the complexes (Figure 4c). Furthermore, the injection of complexes did not result in an increase in the IL-6 level in blood, indicating the low in vivo immunogenicity of complexes (Figure 4d).

## 4. Conclusions

In conclusion, we employed the hBD23 peptide as a platform for in vitro and in vivo mRNA delivery. The positively charged hBD23 peptide could form complexes with mRNA at various ratios. Optimal conditions for the intracellular and in vivo delivery of complexes and subsequent protein expression were established. As the hBD23/mRNA complexes did not show significant cytotoxicity and immunogenicity, we expect that the potential of hBD23 as an mRNA delivery platform could be harnessed for the development of mRNA-based therapeutics.

## Data Availability

The data presented in this study are available on request.
